# Toward a Comprehensive Understanding of Flavor of Sunflower Products: A Review of Confirmed and Prospective Aroma- and Taste-Active Compounds

**DOI:** 10.3390/foods14111940

**Published:** 2025-05-29

**Authors:** Lachinkhanim Huseynli, Christoph Walser, Luise Blumenthaler, Kristel Vene, Corinna Dawid

**Affiliations:** 1 Department of Chemistry and Biotechnology, Tallinn University of Technology, Akadeemia tee 15, 12618 Tallinn, Estonia; lahuse@taltech.ee; 2 Food Chemistry and Molecular and Sensory Science, TUM School of Life Sciences, Technical University of Munich, Lise-Meitner-Strasse 34, D-85354 Freising, Germany; christoph.hald@tum.de (C.W.); luiblum@gmx.de (L.B.); 3 Functional Phytometabolomics, TUM School of Life Sciences, Technical University of Munich, Lise-Meitner-Strasse 34, D-85354 Freising, Germany

**Keywords:** sunflower, taste, metabolites, polyphenols, aroma, *Helianthus annuus* L.

## Abstract

The global demand for sustainable protein sources has led to a growing interest in plant-based alternatives, with sunflower products emerging as a promising yet underutilized option. This review provides a comprehensive overview and critical evaluation of current knowledge on the flavor and off-flavor profiles and codes of sunflower seeds and their by-products, with a focus on both volatile and non-volatile low-molecular-weight compounds. It can highlight the importance of the sensomics approach and the knowledge on key food odorants and tastants. Furthermore, this review underscores the importance of advanced analytical methodologies for linking chemical composition to sensory outcomes. While volatile compounds that activate human olfactory receptors, such as aldehydes, terpenes, and pyrazines, are well described in sunflower products, using the sensomics approach the key odor-active stimuli are just verified in sunflower oil. In addition, the roles of non-volatile components including lipids, proteins, carbohydrates, and secondary metabolites such as polyphenols require further investigation and experimental validation to confirm their role as key tastants and their effect on sensory perception. By compiling existing data, this review establishes a foundational database of known and potential flavor-relevant compounds in different sunflower products, providing a valuable resource to directly or indirectly guide sensory (sensomics) studies and promote sunflower-based product innovation. Identifying the key flavor contributors in the different sunflower-based products and raw materials would facilitate precise approaches in processing and product formulation to enhance sensory quality while mitigating off-flavors. Addressing these challenges will support the development of sunflower-based food products with optimized flavor and nutritional profiles, consistent with global sustainability goals and consumer acceptance.

## 1. Introduction

**Figure 1:**
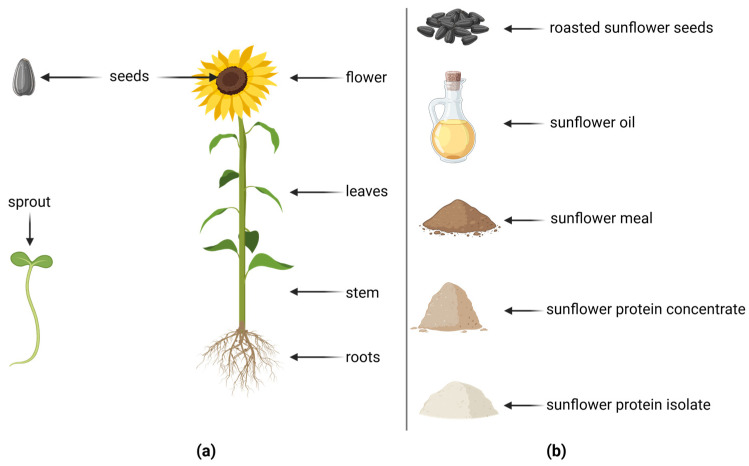
(**a**) Parts of a sunflower plant according to Puttha et al. [13] and (**b**) products derived from sunflower seeds: whole seeds, oil, meal, protein concentrate, and protein isolate. Created with BioRender. Huseynli, L. (2025) https://BioRender.com/okkej50 (accessed on 22 May 2025).

The sunflower (*Helianthus annuus* L.) belongs to the family Asteraceae [1] and has its origins in North America (Figure 1). Sunflowers are cultivated worldwide for their fruits, for human consumption as they provide essential nutrients and as a livestock feed resource [2]. In the context of sunflower, the term “seed” describes the whole fruit, whereas the term “kernel” describes the dehulled seed [3]. According to the United States Department of Agriculture (USDA) [4], sunflower kernels contain approximately 51% fat, 21% protein, and 20% carbohydrates (Table 1).

The sunflower hull accounts for 25–30% of the fruit [2] and often remains unused. It contains 50.0% cellulose and lignin, 25.7% reducing sugars, 4.0% proteins, and 5.17% lipids and wax [5]. The hull has a chemical composition similar to that of sunflower kernels but has a 100-fold lower polyphenol content [6].

Sunflowers are categorized into two main types on the basis of their seed composition. Oilseed sunflower seeds, which contain at least 40% lipids, are used to produce different types of oil such as mid-oleic, high-oleic, high-stearic, and high-palmitic sunflower oil, which have distinct applications. Non-oilseed sunflowers are used for human consumption (in confectionery) or as livestock feed [2]. Factors such as growth conditions, abiotic and biotic stress conditions, and storage practices cause variations in the chemical composition of sunflower seeds, even within the same plant species or genotype [7]. The fatty acid content varies according to the climate and seasonal conditions. For example, higher total lipid content and oleic acid concentrations are observed under warmer conditions. Moreover, processing conditions such as high temperature and high pressure significantly affect the nutritive value of sunflower seeds [8].

Additionally, germination induces changes in the seed chemical composition. For example, sunflower sprouts contain a higher concentration of phenolic acids and flavonoids than ungerminated seeds [7]. Sunflower seeds have a high number of tocopherols and polyphenols, contributing to their significant antioxidant potential, which surpasses that of many other commonly consumed seeds, such as flax, chia, and sesame [9,10]. Tocopherols have a significant anti-inflammatory effect, which alleviates asthma, osteoarthritis, and rheumatoid arthritis. Sunflower seeds also contain several healthy unsaturated fatty acids, proteins, fiber, vitamins, and minerals, which are beneficial for nerves, muscles, bones, and blood in humans. In traditional medicine, sunflower seeds are used to treat cancer owing potentially to their antioxidant effects and rich selenium content, which induce apoptosis of cancerous cells [8,11].

Sunflower sprouts not only have a higher nutritive value but also contain fewer anti-nutritional factors than fresh seeds. Previous research suggests that metabolic changes occur during sunflower seed germination at different temperatures [12]. The findings showed that the concentrations of fatty acid methyl esters and free fatty acids decrease during germination, whereas the concentrations of sterols, α-tocopherols, amino acids, and carbohydrates increase. This metabolic shift occurs as triacylglycerides are broken down and converted to sugars through the glyoxylate cycle, serving as the main energy source for embryonic development. Additionally, a study reported a 22% increase in total polyphenol content, attributed to the activation of endogenous enzymes [12].

The sunflower crop is cultivated mainly to produce oil for human consumption, with the annual global sunflower production reaching 45 million tons, yielding 19 million tons of sunflower oil annually as of 2017/18 [14,15]. The largest producers include Ukraine, Russia, and the European Union, which together dominate the global supply [14]. After palm, soybean, and rapeseed oil, sunflower oil is the fourth most important commercial vegetable oil [1]. Over the years, sunflower yield, oil content, and disease resistance have been optimized through hybridization. These advancements have enhanced not only its agricultural value but also its health benefits. Sunflower oil is reported to reduce blood cholesterol levels because of its low saturated fatty acid content and high levels of oleic acid. Apart from its dietary benefits, the oil is also valued for external use in skin treatments and rheumatism [16].

During oil production, a large quantity of sunflower meal is generated, which consists of proteins, cellulosic fibers, lignins, other polyphenols, and minerals [17]. In 2019, approximately 21.85 million tons of sunflower meal was produced worldwide [18]. Sunflower meal is an inexpensive and rich source of nitrogen and carbon and can be used as a functional food or as an antioxidant supplement [19]. It is mainly used as animal feed and occasionally as fertilizer.

With the rapidly growing population, the requirement for nutrients, especially proteins, is also increasing rapidly; however, the cultivable acreage remains the same. This imbalance between nutrient needs and agricultural capacity threatens several valuable ecosystems [20]. To address this challenge, it may be necessary to adopt novel approaches that optimize the use of by-products from existing agricultural production practices. Sunflower meal, a by-product of oil production, is a rich source of protein (30–34%) [21,22]. However, its protein quality is limited by its amino acid composition, with lysine as the primary limiting amino acid [23,24,25,26,27]. The protein digestibility-corrected amino acid score (PDCAAS) of sunflower protein sources varies across studies but is generally reported as ∼0.6, indicating moderate protein quality [28,29,30,31,32,33]. It can be complemented with lysine-rich proteins such as pea and rapeseed proteins to improve its nutritional value, which enhances its overall amino acid profile [32,34]. A previous study has reported that a 1:1 blend of sunflower and pea protein compensates for limiting amino acids, resulting in a more complete and nutritionally balanced protein profile [34].

A few studies suggest that sunflower proteins require fewer odor-masking agents than pea proteins, making them a more favorable choice in formulations [35]. Additionally, sunflower flour is considered blander than soybean flour, further enhancing its appeal [36]. However, sunflower flour can impart a bitter taste in some food products [37]. Despite its advantages in certain aspects, understanding the taste profile of sunflower products remains challenging.

To promote the use of sunflower as a protein source, it is crucial to identify the off-flavor compounds and pleasant-smelling and pleasant-tasting molecules and to facilitate the broader use of sunflower seeds as a food ingredient by improving their sensory acceptability. The present study aimed to achieve this objective by compiling a database of both volatiles and non-volatiles that have the potential to be aroma- and taste-active compounds in sunflower seeds. In addition, this review highlights methodological and knowledge gaps in flavor research in relation to sunflower-based products.

## 2. Integrated Physiology of Aroma and Taste Perception

The perception of complex food matrices arises from the interplay of gustatory and olfactory stimuli. The human gustatory sense comprises five taste qualities, namely sweet, umami, bitter, salty, and sour, whereas the olfactory cells can detect approximately 10,000 distinct odors [38]. Odor-active volatile compounds are detected by olfactory receptors, whereas taste-active non- or semi-volatile compounds stimulate chemosensory receptors and nerve ends located on the tongue and throughout the oral cavity. Together, these sensory inputs and synergistic interactions contribute to flavor, which is defined as the combination of taste, texture, and odor [39].

The odor compounds play an essential role in assessing the freshness, ripeness, and overall desirability of food. Volatile aroma compounds emitted by ripe fruits signal high energy and nutrient availability, complementing the sweetness detected by taste receptors. Conversely, unpleasant odors from spoiled or fermenting food often indicate microbial activity or toxicity, discouraging consumption [40].

**Figure 2:**
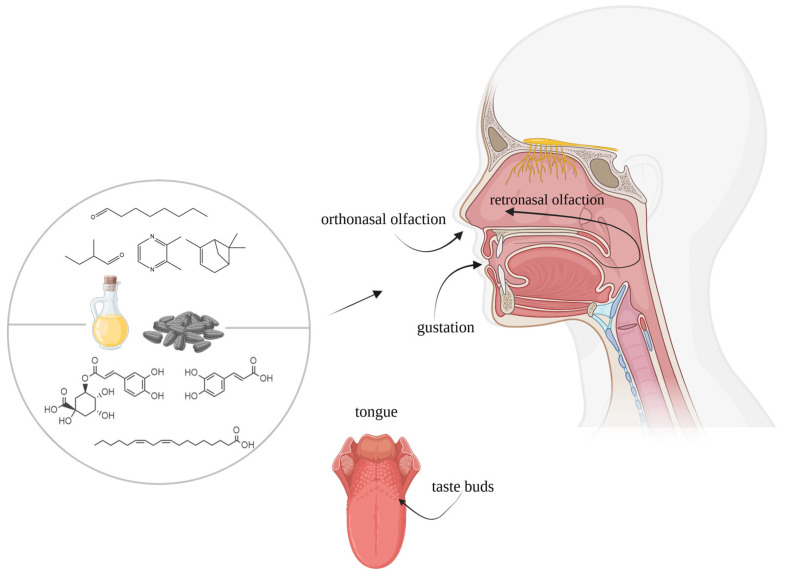
Overview of taste and smell pathways [58] involved in the flavor perception of sunflower-derived products. Created with BioRender. Huseynli, L. (2025) https://BioRender.com/u8c0013 (accessed on 6 May 2025).

Aroma perception begins when volatile molecules enter the nasal cavity and bind to the ~390 odorant receptor proteins on sensory neurons located in the *regio olfactoria*. These neurons detect aroma stimuli that enter the nasal cavity during inhalation (orthonasal) or are released from food during consumption (retronasal). The binding and interaction of these molecules to specific olfactory receptors are determined by their molecular structure and concentration, triggering neural signals that are processed in the olfactory bulb and higher brain centers [41]. These signals, in combination with gustatory inputs, modulate taste perception by enhancing, suppressing, or complementing specific taste attributes, which significantly shapes the overall flavor experience (Figure 2).

The identification of volatile aroma-active compounds involves specialized techniques that differ from those used to identify taste-active compounds. One such method is gas chromatography–olfactometry (GC-O), which combines gas chromatographic separation with human sensory detection to identify and quantify aroma-active compounds. In GC-O, volatile compounds are separated chromatographically and then assessed by trained panelists who sniff the effluent to detect odor-active components [42]. Although GC-O facilitates the detection of odor-active volatiles, it does not determine which compounds are truly essential for flavor perception. Many of the detected compounds may be present in concentrations too low to influence the sensory experience, whereas others may interact in ways that amplify or suppress their effects. The sensomics approach may be used to identify the key aroma-active and taste-active compounds responsible for sensory characteristics. Unlike conventional profiling methods that simply list the detected compounds, sensomics combines sensory-guided fractionation with analytical techniques to identify the key flavor-active compounds in complex food matrices [43]. By directly linking sensory perception to chemical composition and to quantitative data via reconstitution and omission experiments, this approach characterizes compounds responsible for specific taste and odor attributes, offering crucial insights into the molecular interactions that define flavor [43,44]. The sensomics approach has been successfully applied to various foods in previous studies [45,46,47,48,49,50].

Taste perception helps animals, especially omnivores with a broad food spectrum, to assess and choose their food [51]. Sweetness, mainly evoked by sugars and some amino acids, indicates a high-energy food source [51]. Similarly, the umami taste serves as a key indicator of protein-rich foods, guiding the selection of nutrient-dense meals [52]. In contrast, bitterness often acts as a warning sign, suggesting the presence of potentially harmful substances such as cyanides and alkaloids, leading to food rejection [51]. However, not all bitter compounds are avoided; some bitter compounds, such as those found in coffee, beer, and red wine, are not only tolerated but even preferred [51,53,54]. This acceptance varies among individuals owing to genetic differences in bitter taste perception and among compounds on the basis of receptor activation affinities [55,56,57]. Salty taste plays a crucial role in maintaining the ion balance in the body, making salty foods necessary for physiological function, whereas sour taste is often associated with unripe fruits, signaling a lower energy content and leading to avoidance in many cases [51].

In general, taste perception is induced by the interaction of taste-active compounds with ~40 taste receptor proteins on the tongue. These receptors are located in specific cells called taste receptor cells. A taste receptor cell expresses receptors specific for only one taste quality. Approximately 50–100 of these taste receptor cells are arranged in taste buds, which are located in taste papillae on the tongue, the soft palate, and the throat [59,60,61,62]. The binding of taste-active molecules to these receptors triggers the activation of specific receptor families, primarily G-protein-coupled receptors (GPCRs) and ion channels, which initiate signaling cascades leading to neurotransmitter release. Once activated, these receptors mediate distinct taste qualities. Sweet taste perception is facilitated by the T1R2/T1R3 GPCR complex, which belongs to the class C GPCR family. These receptors detect a broad range of sweet stimuli, including sugars, artificial sweeteners, and certain amino acids. Upon ligand binding, the receptor undergoes a conformational change, activating intracellular signaling pathways that result in neurotransmitter release and subsequent taste perception [60,63]. In addition to T1R2/T1R3, other mechanisms have been reported to be involved in sugar detection, providing an alternative pathway for sweet taste transduction [63,64,65]. Similarly, the umami taste, which signals the presence of amino acids and nucleotides, is primarily mediated by the T1R1/T1R3 receptor complex. This receptor is specifically responsive to L-glutamate and synergistically activated by 5’-ribonucleotides such as inosine monophosphate (IMP) and guanosine monophosphate (GMP). Additionally, metabotropic glutamate receptors (mGluR1 and mGluR4) are also involved in umami perception, particularly for detecting free glutamate at dietary concentrations [63,65,66]. These receptors activate G-protein-mediated signaling pathways, leading to intracellular calcium mobilization and neurotransmitter release. Bitter taste perception, in contrast, is facilitated by a diverse family of T2R GPCRs, with approximately 25 functional receptors in humans. Each T2R can recognize multiple structurally diverse bitter compounds, and conversely, many bitter compounds can activate multiple T2Rs [59,60,63,67,68]. Upon activation, the T2Rs initiate intracellular signaling cascades via the G-protein gustducin, which ultimately leads to neurotransmitter release and aversive taste perception [66,69]. Notably, genetic variations in T2Rs contribute to individual differences in bitter taste sensitivity [59]. Remarkably, sour taste is primarily mediated by intracellular acidification rather than extracellular proton concentration. Weak organic acids, such as citric acid and acetic acid, penetrate taste cells and lower the intracellular pH, leading to inhibition of potassium leak channels and subsequent depolarization of the cell membrane [63]. Type III taste cells have been identified as the primary detectors of sour stimuli, and recent evidence suggests that inwardly rectifying potassium channels play a key role in mediating the sour taste response [66]. Finally, salty taste transduction remains less well understood, but it is known that epithelial sodium channels are crucial in detecting low concentrations of sodium. These amiloride-sensitive channels allow sodium ions to enter the cell, leading to membrane depolarization and neurotransmitter release [60]. However, high concentrations of sodium are detected via an amiloride-insensitive pathway, likely involving other ion channels [63]. Unlike the other taste modalities, which rely on GPCRs, salty taste perception is mediated by direct ion flux through channels [66]. 

In general, the activation of taste receptor cells leads to neurotransmitter release, conveying sensory information via afferent nerve fibers to the brain, where it is processed and integrated with other sensory inputs to produce the perception of taste [59,60,61,63,66].

Four attributes are used to describe taste perception: (i) quality, which is the most important sensation for defining taste; (ii) intensity, which describes the magnitude of the sensation; (iii) temporal pattern, which characterizes how long the taste perception lasts; and (iv) spatial topography, which explains the location of taste perception [70]. Similarly, odor perception includes attributes such as intensity, hedonic tone, and aroma character. An important indicator is the recognition threshold of a taste stimulus, which describes the lowest concentration of a taste-active compound that can be perceived as a specific taste [71]. This threshold is determined using three-alternative forced choice test or a half-tongue test by a qualified sensory panel [72]. To further assess a compound’s contribution to sensory perception, the dose-over-threshold (DoT) factor is commonly used. This factor is calculated as the ratio between a compound’s actual concentration in a food matrix and its sensory detection threshold, where a DoT value above one indicates that the compound contributes to taste perception [73,74,75,76,77].

Furthermore, somatosensory perceptions modify taste perception and contribute to flavor. These include sensations such as pungency, which arises from the activation of the transient receptor potential vanilloid receptor, as well as chemical-induced cooling and astringency, both of which influence flavor perception. The sensory perception of a prickle is characterized by a mild tingling sensation, often experienced as a light electric-like stimulation on the tongue, which may be associated with mild irritation or discomfort. Similarly, orosensory detection of CO_2_ and the somatosensory perception of fats through specific receptors and nerve endings shape flavor perception. Finally, the concept of kokumi perception, which is exhibited by compounds modulating and intensifying the perception of all flavors, is important for a food’s flavor profile [78].

## 3. Characterization of Volatile Compounds in Sunflower Products and Their Potential Role in Aroma Formation

The aroma profile of sunflower products is shaped by a complex interplay of volatile compounds, influenced by processing methods, chemical transformation, and differences in species or cultivars. Understanding these factors is essential for optimizing the sensory quality and addressing potential off-flavors. Numerous studies have explored the volatile and aroma profiles of sunflower products, but only one has used a molecular sensory science approach to identify key odor-active compounds in sunflower oil [79]. However, this comprehensive methodology has not yet been extended to other sunflower-derived products, limiting the precise identification of their key odor-active compounds.

According to previous studies [80,81], raw sunflower seeds contain dominant volatiles such as terpenes, aldehydes, lipid oxidation products; among these, α-pinene is prominent for its distinct pine-like aroma, contributing to the overall sensory profile. Other major volatiles in raw seeds include hexanal, furfural, octane, and γ-butyrolactone, which impart grassy, caramel, and other nuanced notes [82]. However, the volatile profile of sunflower seeds undergoes significant changes during roasting, primarily through the Maillard reaction and enhanced lipid oxidation. α-pinene, a major volatile in raw sunflower seeds, remains dominant in roasted seeds, exceeding its odor threshold and potentially contributing to a pine-like aroma [79,80]. Similarly, several volatiles, including 2-methylbutanal, 3-methylbutanal, and 1-octen-3-ol, are present in both raw and roasted seeds; however, their concentrations often increase with roasting. Pyrazines, such as 2-ethyl-3-methylpyrazine, 2,5-dimethylpyrazine, 2,3-dimethylpyrazine, and 2-ethyl-3,5-dimethylpyrazine, dominate the roasted aroma, contributing nutty and roasted notes. During roasting, furfural, a furan derivative known for its caramel-like aroma, increases alongside other lipid oxidation products such as pentanal and nonanal. These compounds enhance the roasted profile, whereas higher concentrations of hexanal, nonanal, and pyridine lead to undesirable off-flavors, particularly at high roasting temperatures and extended heating periods [79]. This characterization of sunflower seeds is based on the flavoromics approach, which uses headspace solid-phase microextraction (HS-SPME) coupled with gas chromatography–mass spectrometry (GC-MS) and principal component analysis (PCA) [81,82]. These studies provide valuable insights into changes in volatile composition; however, they do not establish a direct correlation between volatiles and sensory perception. At the same time, certain volatiles have been reported to exceed their odor thresholds, suggesting they contribute to the aroma profile, but no recombination or omission tests were conducted to confirm their actual impact on perception. Without such validation, the role of these key odorants remains speculative. Especially highlighted by the groups of Thomas Hofmann and Peter Schieberle, key food odorants have to be defined via reconstitution and omission tests to verify the importance of single flavor stimuli in a complex food system [83]. Additionally, the methods used for volatile extraction may not fully capture all aroma-active compounds; instead, using solvent-assisted flavor evaporation (SAFE) [43] could offer a broader and more representative volatile profile by leading to a more comprehensive understanding of sunflower seed aroma.

Regional practices and seed varieties influence the volatile profile, as seen in Chinese traditional aromatic sunflower seeds, which feature unique compounds such as eugenol, *E*-anethole, and *E*-cinnamaldehyde, known for their spicy and floral notes [84]. A previous study identified volatile compounds in Chinese traditional aromatic sunflower seeds using static headspace and simultaneous distillation and extraction methods, followed by GC-MS analysis [84]. Although odor activity values (OAVs) were calculated to assess aroma contribution, the absence of a recombination test limits the confirmation of the role of these compounds in aroma perception [43].

Although sunflower oil is extracted from sunflower seeds, its volatile profile evolves uniquely through extraction, processing, and storage, reflecting both similarities with and differences from the raw material. These changes lead to a diverse array of aroma-active compounds that define the sensory identity of sunflower oil.

Cold-pressed sunflower oil is widely recognized for its distinctive volatile profile, with α-pinene consistently identified as the dominant compound. Bocci et al. (1996) [85] highlight that α-pinene accounts for most of the volatile fraction, accompanied by other terpenes such as limonene, sabinene, *β*-pinene, and 1,2,6,6-tetramethyl-1,3-cyclohexadiene. Additionally, minor quantities of hexanal and traces of unidentified volatiles, likely other terpenic hydrocarbons, have been reported using dynamic headspace sampling with GC-MS. Considering the study’s focus on volatile composition rather than aroma perception, the absence of sensory validation methods reflect methodological limitations during that period [85].

According to Bendini et al. [86], sunflower seed-like and nutty aromas in virgin or cold-pressed sunflower oils are linked to α-pinene and 3-methyl-1-butanol, identified as volatile markers through solid phase microextraction–gas chromatography (SPME-GC) and quantitative descriptive analysis (QDA) [86]. In contrast, samples exhibiting defects, such as rancid or fried oil off-notes, showed elevated levels of E-2-heptenal, a volatile aldehyde indicative of lipid oxidation. However, the absence of direct sensory validation techniques, such as GC-olfactometry (GC-O) analysis and aroma recombination experiments, limits the ability to confirm the aroma contribution of these volatiles.

**Figure 3:**
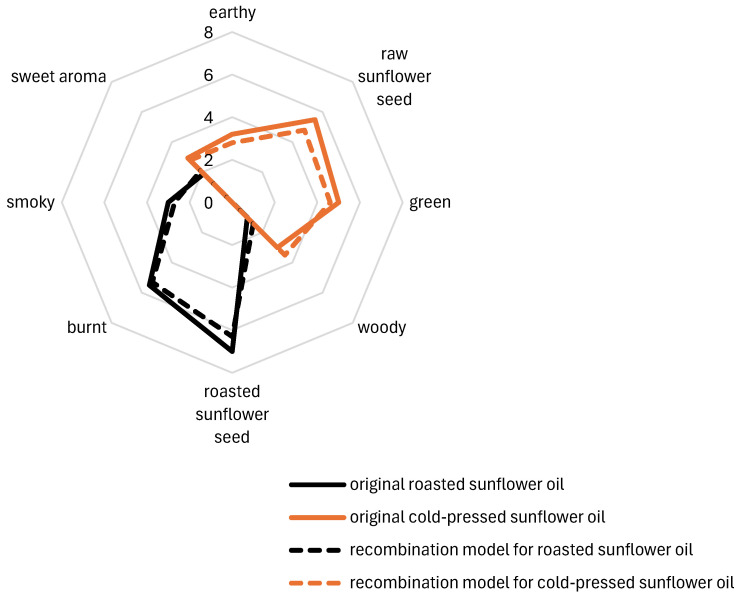
Aroma profiles of cold-pressed and roasted sunflower oils and their corresponding aroma recombination models (adapted from [79]).

Yin et al. [79] provide further insight into the key odor-active compounds in cold-pressed sunflower oil using molecular sensory science which offers comprehensive analysis through techniques such as SAFE and aroma extract dilution analysis and emphasizing the role of volatiles with high OAVs. The researchers were able to rebuild recombination models with no significant differences from the original samples (Figure 3). This analysis identified α-pinene, *β*-pinene, linalool, hexanal, octanal, α-phellandrene, and (*E*)-2-octenal as the most significant contributors to the sensory profile of sunflower oil. These compounds collectively define the raw sunflower seed, woody, green, earthy, and sweet aromas characteristic of cold-pressed oils. In contrast to cold-pressed oil, roasted sunflower oil undergoes significant changes during heat processing, resulting in a more complex and intense aroma profile. Thermal degradation of terpenes and the promotion of Maillard reaction pathways lead to the formation of compounds such as pyrazines, furans, and aldehydes, which dominate the aroma of roasted oils. As a result of these reactions [78], the key contributors to the roasted, smoky, and burnt aromas include 2- and 3-methylbutanal, 2,6-dimethylpyrazine, 2,5-dimethylpyrazine, 2,3-dimethylpyrazine, 2,3-pentanedione, 2-pentylfuran, 2,3-dimethyl-5-ethylpyrazine, and 1-pentanol [79].

Processing methods such as microwave treatment enhance the roasted and smoky aromas while reducing the raw sunflower seed and woody notes, as demonstrated in a previous study using SAFE, HS-SPME, and GC-O-MS [87]. This study highlights changes in aroma-active compounds; however, it does not include OAVs, which are essential for understanding the impact of these compounds on the overall aroma profile [43]. Study [87] also mentions that enzymatic treatments promote the release of aroma precursors, leading to the formation of compounds such as 2,3-dihydro-3,5-dihydroxy-6-methyl-4H-pyran-4-one, benzeneacetaldehyde, furaneol, and acetic acid. In addition to enhancing sensory richness, these treatments improve oxidative stability by lowering the acid and peroxide values, offering dual benefits [87].

Analysis of Chinese sunflower oil using HS-SPME-GC-Quadrupole-MS, along with QDA and partial least squares regression, revealed that specific volatiles are positively correlated with sensory notes. For example, the “ripe sunflower seed” note was linked to 1-octen-3-ol, *n*-heptaldehyde, and dimethyl sulfone; the “sunflower seed” attributes were associated with γ-terpinene, octanal, and (±)-linalool; and “sweet” notes were derived from aldehydes such as *n*-nonanal and 2,3-butanediol [88]. However, although these correlations provide valuable insights, the study relies on statistical associations and literature descriptors rather than direct sensory validation methods, which strengthen the confirmation of aroma-active compounds (Table 2).

In conclusion, a systematic sensomics approach for a more comprehensive analysis of sunflower seeds remains lacking. Implementing a sensomics approach step by step plays a critical role: dilution analyses help narrow down potent odorants; quantification allows concentration-to-threshold comparisons; and omission/recombination tests validate whether these compounds truly shape perception. Without applying this approach, findings remain partial and risk misidentifying or overlooking key contributors to flavor. As a result, conclusions about sensory quality and potential improvements may be speculative or misleading.

Additionally, the understanding of key aroma compounds in sunflower-derived protein sources such as meal, concentrates, and isolates remains limited. Addressing their sensory challenges is essential as these ingredients receive increasing attention for food applications.

## 4. Mapping Non-Volatiles and Taste-Associated Metabolites in Sunflower

Although it is critical to study the volatile and potentially aroma-active compounds that contribute to the characteristic sensory profile of sunflower products [79,81,90], it is equally important to consider the potential role of non-volatile compounds in taste perception. Unlike volatiles, non-volatile and semi-volatile compounds influence taste directly by interacting with ~40 taste receptors, contributing to taste attributes such as bitterness or sweetness [60,63,66]. However, the specific contribution of these compounds to the taste of sunflower seeds has not yet been experimentally validated using sensomics approaches.

Plant phytochemicals, the sources of many sensory attributes, are broadly categorized into primary and secondary metabolites. Primary metabolites such as sugars, organic acids, amino acids, and nucleic acids are essential for plant growth and metabolic functions. In contrast, secondary or special metabolites, which are classified on the basis of their chemical structure, play a non-essential yet critical role in plant survival as defense-related compounds. These compounds protect against environmental stressors such as microbes, herbivores, and UV radiation and contribute to sensory properties such as bitterness and astringency. Additionally, they aid in attracting pollinators and influence human sensory perception such as bitterness and astringency, which stem from their interaction with taste receptors [91].

**Figure 4:**
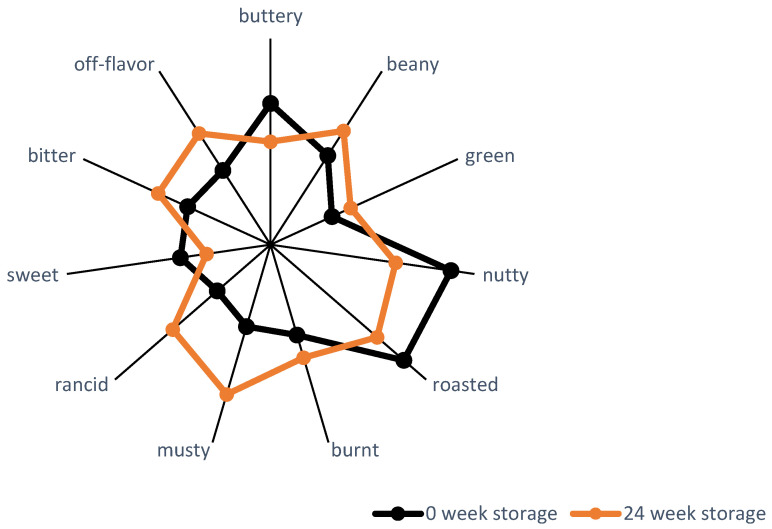
Taste profiles of roasted sunflower kernels at 0 and 24 weeks of storage adapted from Robertson et al. [92].

The sensory profile of freshly roasted sunflower kernels is defined by nutty, roasted, buttery, and sweet attributes. However, over time, storage-related changes result in the development of off-flavors, such as unpleasent off-notes. This dynamic sensory evolution, as documented in a study from 1988, is captured in Figure 4 [92]. More recent studies have explored sensory evaluation in terms of overall liking or acceptance, but they often lack the depth required to identify and analyze specific sensory attributes [93,94,95]. This gap underscores the need for updated research.

To further understand the potential contributors to sunflower seed flavor, it is essential to consider the role of macronutrients and their potential effects on taste perception through interactions with taste receptors and other sensory-active compounds. The following section explores the macronutrient and micronutrient composition of sunflower products, highlighting their presence, distribution, and relevance.

Among the reported non-volatile compounds, the approaches used for their identification and quantification differ widely across studies. These methodological differences should be taken into account when interpreting the values presented in the upcoming tables. Detailed analytical procedures can be found in the corresponding original publications referenced in each table.

### 4.1. Macronutrients

#### 4.1.1. Lipids

The main fraction of sunflower seeds corresponds to lipids (35–42%), primarily in the form of triacylglycerides, which show high variability in their fatty acid composition owing to varietal differences; nevertheless, triacylglycerides such as 50:2, 52:3, 52:4, 54:5, and 54:6 have been reported to be the most abundant in sunflower seeds [96,97]. A characteristic feature of sunflower seeds is the presence of very-long-chain fatty acids, such as arachidic acid and behenic acid. Although the seeds contain only small quantities of free fatty acids, these compounds significantly influence specific flavors and promote fat oxidation. The free fatty acid content depends on the enzymatic activity of lipase, which hydrolyzes triacylglycerides into free fatty acids. This activity is further accelerated by wet harvesting and moisture during storage [98]. Furthermore, as mentioned earlier, sunflower seeds are broadly classified into non-oilseed and oilseed varieties, with oilseed varieties being highly diverse in their fatty acid profiles, suited for distinct industrial and sensory applications [99,100,101]. These differences in the composition of fatty acids may significantly affect their flavor contribution and the characteristics of their oxidation products. The oxidation reactions contribute to off-flavors and reduce the nutritional value of the seeds. Table 3 [102,103] shows the fatty acid composition of sunflower seeds. In the referenced study, fatty acids were extracted using the Bligh and Dyer method, methylated with acid catalysis, and analyzed by GC-FID using nonadecanoic acid (C19:0) as the internal standard [103]. Please note that different studies may use varying extraction and analytical methods, which can lead to differences in reported values.

Roasted sunflower seeds contain palmitoleic acid (16:1), eicosanoid acid (20:0), and gadoleic acid (20:1) [104]. Typically, fresh, intact sunflower seeds contain low concentrations of linoleic acid, *α*-linolenic acid, oleic acid, palmitic acid, and stearic acid; however, variations in composition may influence their potential contribution to taste perception [12,103]. However, during storage or processing methods such as soaking, the fatty acid composition of germinated seeds changes. These alterations influence the sensory perception of sunflower seeds by modifying sensory attributes, potentially leading to rancid, musty, bitter, and beany flavors [92].

Sunflower oil is rich in unsaturated fatty acids, particularly the essential omega-6 fatty acid linoleic acid, which comprises 55–70% of the lipid fraction [1]. The oil contains only 1.19% –1.35% (*w*/*w*) free fatty acids [98]. These free fatty acids, despite their low concentrations, are associated with a bitter taste, which is generally disliked [51]. To mitigate oxidation and maintain nutritional value, the presence of antioxidants such as phenolic acids is crucial. However, sunflower oil contains phenolic acids only in trace amounts owing to their poor solubility in oil [6,105].

A previous study reported DoT values of >1 for certain fatty acids (Table 3), indicating that they contribute to taste perception. Of these fatty acids, linoleic and oleic acids exhibited higher DoT values, suggesting a potentially more significant role in taste modulation. This is consistent with their predominance among fatty acids in sunflower oil [106,107]. Notably, these values vary with processing conditions and seed fatty acid composition.

#### 4.1.2. Proteins

Sunflower seed protein is increasingly being explored for its role in plant-based formulations. In particular, sunflower meal and press cake represent a promising alternative protein resource in food applications, consistent with efforts to diversify protein sources [108]. Sunflower seeds contain approximately 20% proteins, primarily the seed storage proteins 11S globulins and 2S albumins. The seed amino acid composition is balanced, with a high proportion of sulfur-rich amino acids, but a notable drawback is the low level of the essential amino acid lysine [1]. The quantity of free amino acids is important for sensory analysis because they impart intrinsic taste activity. For example, histidine, isoleucine, leucine, phenylalanine, and valine, in sufficiently high concentrations, impart a bitter taste [109,110,111,112,113]. In contrast, alanine, glycine, serine, threonine, and proline are sweet-tasting amino acids, whereas glutamic acid and aspartic acid are associated with an umami note when present in concentrations above their threshold [110,111,112,113,114].

Data on the amino acid content of sunflower seeds are limited, and when presented, they are often shown in charts, making it difficult to determine the exact numerical values [12,115]. However, several studies have investigated the amino acid composition of sunflower meal, isolate, hydrolysates, and press cake [26,116,117,118,119]. This is particularly important because these products are protein alternatives, and understanding their composition is crucial for assessing their potential applications and addressing flavor challenges. A previous study [117] reported specific numerical data for certain amino acids in sunflower seeds (Table 4). Although it does not provide data for all amino acids, it offers valuable insights into their composition. Study [117] demonstrated that sunflower oilcakes have a higher total amino acid content than seeds. In descending order, alanine, glycine, glutamic acid, leucine, and aspartic acid were reported to be the most abundant amino acids in seeds. Asparagine and glutamine were likely not detected because they are more likely converted into aspartic acid and glutamic acid, respectively, during acidic hydrolysis. Seeds contain a total amino acid content of 5790.26 nmol/g, with 6.63% being essential and 93.37% non-essential amino acids. However, seeds exhibit fewer detectable amino acids than meal products, such as valine, isoleucine, methionine, and tryptophan, among others, are absent because of the protective role of hulls, which reduce enzymatic digestibility [12,115,117,120]. The presence of tryptophan, asparagine, and glutamine was also not reported by Chen et al. [115].

According to Bao et al. [121], enzymatic hydrolysis of sunflower seed proteins with Flavourzyme generated umami-tasting peptides, with the highest umami intensity observed after 480 min of hydrolysis performed using an electronic tongue. At this point, peptides such as DVNNPANQLD, NNENQLDEYQR, and EFEGGSIEH were identified as contributors to the umami taste. However, prolonged hydrolysis increased bitterness, likely owing to the release of bitter-tasting amino acids and hydrophobic peptides, highlighting the importance of regulating the degree of hydrolysis to optimize the flavor balance. These findings suggest potential applications of sunflower seed hydrolysates as natural flavor enhancers in plant-based or low-sodium food products. Among the reported amino acids in Table 4, only glutamic acid exhibited a DoT value >1, signifying its contribution to the umami taste. Furthermore, products of Maillard reaction, whereby the carbonyl group of reducing sugars reacts with an amino group, affect the flavor, color, and stability of sunflower seeds [122]. A previous study reported that the Maillard reaction promoted by heating and addition of xylose and cysteine to sunflower peptides intensifies the meat-like flavor and umami taste [123]. The Maillard reaction may occur in sunflower seeds naturally or during processing.

#### 4.1.3. Carbohydrates

In sunflower kernels, the predominant carbohydrates are complex polysaccharides and fiber, with their reported content ranging from 2.86 to 3.88 g/100 g. The total carbohydrate content in sunflower seeds varies across studies, reported between 14.72 and 27.36 g/100 g, influenced by the variety and analytical methods used [124]. According to Muttagi et al. [124], the total sugar content of sunflower seeds ranges from 2.36 to 3.04 g/100 g, consistent with the values reported by the USDA [124,125,126].

Similarly, Laemont et al. [80] reported that the total sugar content in sunflower seeds is relatively low, comprising approximately 3%, with sucrose being the predominant sugar at 3.2 g per 100 g. During roasting, sucrose gradually breaks down into fructose and glucose, and significant changes occur in carbohydrate composition, potentially influencing flavor development. The sugar content of sunflower seeds fluctuates during germination as well [12,80].

The starch content of sunflower seeds is notably low (0.42%) [2]. In contrast, sunflower hulls are primarily composed of structural carbohydrates, with cellulose and lignin together constituting nearly 50% of the hull weight. Reducing sugars represents the second largest component, comprising 25.7% of the total hull weight. This compositional distinction underscores the role of sunflower kernels as a nutrient-rich storage unit, whereas the hull provides essential structural support to the seed [5].

In sunflower seeds, free monosaccharides such as fructose, glucose, galactose, and mannitol are found in low concentrations below their threshold values. Fresh sunflower seeds also contain small quantities of arabinose and trace amounts of rhamnose [127].

Given that the reported sucrose content in sunflower seeds is 3.2 g/100 g (93,485 µmol/kg) [80], surpassing its taste detection threshold of 12,500 µmol/kg [128], (DoT~7) suggests that sucrose is likely to contribute to the overall taste perception. However, the slight sweetness of sunflower seeds likely arises from the combined effect of multiple sweet-tasting molecules.

### 4.2. Micronutrients

#### 4.2.1. Minerals and Vitamins

Sunflower seeds contain valuable nutrients, vitamins, and minerals [1]. Among these, sodium and potassium ions are particularly associated with salty tastes, with intrinsic taste thresholds of 3900 µmol/kg and 13,000 µmol/kg, respectively [46]. In some foodstuffs, minerals such as magnesium and calcium potentially contribute to bitter and astringent taste perception, in addition to salty taste [46]. A recent study [125] provided a detailed analysis of the mineral content of dehulled sunflower seeds, revealing higher levels of minerals in dehulled seeds than in whole seeds owing to the removal of mineral-poor hulls. For instance, dehulled seeds contained iron (6.4–10.19 mg/100 g), zinc (9.533–17.855 mg/100 g), magnesium (8.21–18.06 mg/100 g), and potassium (5.14–7.0 mg/100 g). These findings differ from the USDA-reported average values for raw sunflower seeds: 302 mg/100 g for magnesium and 657 mg/100 g for potassium [126]. The study attributed these discrepancies to differences in sunflower hybrids, climatic conditions, soil quality, and the USDA’s reliance on averaged data across multiple sources. These differences can potentially alter the flavor profile.

To provide context, the mineral levels shown in Table 5 [2,46,126] reflect USDA values for raw sunflower seeds. DoT values calculated on the basis of these values suggest that minerals such as magnesium and potassium play a notable role in sunflower seed taste perception.

In addition, sunflower seeds are an excellent source of B vitamins and the antioxidant vitamin E. The high concentration of vitamin E (87,762 µmol/kg) helps to inhibit photooxidation [1]. Most vitamins are taste-active compounds, and sunflower seeds contain approximately 6.96 mg of niacin, 0.84 mg of pantothenic acid, 0.76 mg of pyridoxine, 0.507 mg of riboflavin, and 0.473 mg of thiamine per 100 g [4,110].

Thiamine (a common flavor precursor), pyridoxine, and pantothenic acid impart an intrinsic bitter taste, whereas niacin and riboflavin are particularly bitter. Thiamine and vitamin C activate sour taste receptors, whereas vitamin K (phylloquinone) exhibits a moderately sweet taste. However, the taste thresholds for these vitamins have not yet been determined [110]. The fat-soluble vitamins A, D, and E are abundant in sunflower seeds; they have traditionally not been associated with significant taste activity [4,129]. Nevertheless, a recent study [130] has shown that vitamins A and D can activate specific bitter taste receptors in vitro, indicating potential taste activity despite earlier assumptions. The same study also reported, for the first time, human bitter taste detection thresholds for vitamin B1 (as thiamine hydrochloride), B2 (as riboflavin phosphate), B3 (as niacinamide), and B6 (as pyridoxine hydrochloride) [130]. As these thresholds were determined using specific supplemental forms of the vitamins, no further calculations were performed to avoid a potentially misleading interpretation.

#### 4.2.2. Phenols

Phenols are compounds characterized by an aromatic ring with at least one hydroxyl group attached to the benzene ring [131]. Approximately 463 phenolic compounds have been identified in sunflower, flaxseed, poppy, pumpkin, and sesame seeds [132]. In sunflower seeds, the total phenolic content in defatted non-oilseed kernels (3291–3611 mg/100 g dry weight) is similar to that in oilseed kernels (3938–4175 mg/100 g); however, the latter exhibit slightly but not significantly higher levels [6]. Chlorogenic acid (5-*O*-caffeoylquinic acid) is the predominant phenol in sunflower oilseeds, with a concentration of 69,628.29 µmol/kg dry weight. Additionally, 3-*O*- and 4-*O*-caffeoylquinic acids are also present [2]. These compounds themselves do not taste bitter, but bitter-tasting derivatives such as chlorogenic acid lactones and caffeoyl quinides may be produced during processes such as roasting [133]. Moreover, these phenolic compounds interact with proteins, potentially lowering the nutritional quality by altering the organoleptic properties and reducing protein digestibility [134].

Furthermore, phenols may undergo browning reactions, during which chlorogenic acid is oxidized to form a highly reactive *o*-quinone intermediate. This intermediate reacts with the amino group of lysine or the thiol group of cysteine or methionine, leading to the development of green or brown discoloration. Such reactions produce toxic compounds, destroy essential amino acids, and reduce the nutritional quality, as the condensation products generated cannot be metabolized. This is one of the primary reasons sunflower protein isolates are not yet widely used in industrial food products [135]. Studies have attempted to address these challenges; for example, dephenolization of sunflower meal by washing the dried meal with 60% methanol for 8 h has been shown to reduce the adverse effects of phenols [22].

Polyphenols exhibit antiviral, antimicrobial, and antioxidant activity, which explains why the total phenolic content in plants increases under mechanical stress [136]. The flavonoid myricetin, found in sunflower seeds, is an even stronger antioxidant than α-tocopherol [137]. In addition to these benefits, phenols are reported to have anti-inflammatory, anticancer, and antimutagenic effects in humans, reducing the risk of coronary heart disease [136]. Phenols are also present in other parts of the sunflower plant; for example, flavonoids found in trichomes act as a natural defense mechanism against insects [138].

Phenolic acids are present in high concentrations in sunflower seeds and are associated mainly with bitterness or astringency. Astringency is described as a puckering and drying sensation on the whole tongue surface, which is linked to phenolic compounds with at least two hydroxyl groups in 1,2-dihydroxy or 1,2,3-trihydroxy configurations. These configurations enhance the binding of phenols to salivary proteins, forming phenol–protein complexes that precipitate when sufficiently hydrophobic, resulting in the characteristic astringent sensation [136]. Table 6 lists the phenols reported in sunflower seeds [2,7,102,137,139,140,141,142,143]. Sunflower oil contains only small amounts of vanillic and p-hydroxybenzoic acids owing to the poor solubility of phenolic compounds in oil [6,142], with reported concentrations of 6.9 μg and 1.5 μg per 100 g, respectively [142].

The phenolic composition of sunflower seeds varies depending on factors such as extraction method, plant species, and growth environment. This has been observed in studies showing that drought stress can lead to higher total phenolic content and antioxidant activity in sunflower seeds [144]. Genotype and cultivation location have also been shown to contribute to significant variations in the phenolic profiles across sunflower varieties [145]. For example, one study [2] reported 638.58 µmol/kg ferulic acid, whereas Žilić et al. [137] reported 272.94–308.99 µmol/kg ferulic acid. Such variability in reported phenolic compound concentrations may alter their DoT value, consequently modulating their sensory impact and direct role in taste perception. Furthermore, synergistic interactions between phenols in sunflower seeds influence taste perception. For instance, ferulic acid (90 ppm) combined with p-coumaric acid (40 ppm) has a bitter taste threshold of 25 ppm [146]. Remarkably, ferulic acid alone has been reported to evoke a sour taste with a threshold of 90 ppm [147].

Furthermore, organic acids such as hydroxyacetic acid, 4-hydroxybutyric acid, phosphoric acid, and 2-piperidinecarboyxlic acid have been detected in fresh and roasted sunflower seeds, but their concentrations have not been reported [104]. Among these, malic acid is characterized by its sour taste, with a threshold of 3700 µmol/kg, whereas ß-aminoisobutyric and γ-aminobutyric acids contribute exclusively to an astringent sensation, with thresholds of 120 µmol/kg and 20 µmol/kg, respectively [104,128,148].

The taste thresholds of flavonoids, a subgroup of polyphenols, in sunflower seeds are presented in Table 7 [7,71,128,137,148,149,150,151]. Among flavonoids, apigenin, kaempferol, and quercetin are present only in small quantities [7]. The quantity of myricetin (131.98–194.82 µmol/kg) in some samples has been reported to exceed the threshold and is more likely to contribute to an astringent and bitter taste. The human tongue is highly sensitive to rutin, a glycoside of quercetin with glucose, which imparts astringency at a detection threshold of only 0.00115 µmol/kg [131,152].

Additionally, sunflower seeds contain other flavonoids such as heliannone and luteolin. The total isoflavone content in sunflower seeds has been reported to be 534 mg/100 g [1]. Isoflavones such as formononetin, daidzein, glycitein, coumestrol, and genistein are also present [149]. Although these compounds are taste-active, their concentrations in sunflower seeds are low. For example, genistein activates the bitter receptors hTAS2R14 and hTAS2R39 at concentrations of 4 µmol and 8 µmol, respectively [150]. In addition, minor lignan components such as matairesinol, lariciresinol, pinoresinol, and secoisolariciresinol are also found in sunflower seeds [149].

Catechins are derived from flavan-3-ol, and some of these elicit a bitter and puckering sensation. (−)-epigallocatechin, (+)-catechin, (−)-epicatechin, (−)-epigallocatechin gallate, and (−)-epicatechin gallate are known to be present in sunflower seeds, but exact concentrations have not been reported [148]. The catechins constitute 0.7 × 10^−3^% of the flavonoids in crude sunflower oil.

The DoT values of most flavonoid compounds based on their reported concentrations in sunflower seeds were below one, suggesting that they do not directly contribute to taste perception, except myricetin, the DoT value (>1) of which indicated a potential impact on taste. These values may vary across sunflower cultivars, influencing overall sensory characteristics.

#### 4.2.3. Other Secondary Metabolites

Bioactive phytosterols are present in higher amounts in sunflower kernels (131.9–511.9 mg/100 g) than in several other nuts and seeds [2]. However, the taste activity of compounds such as *β*-sitosterol, campesterol, stigmasterol, and 7-stigmastenol has not been reported. Additionally, sunflower seeds contain various lignans, including 1-acetoxypinoresinol, 7-hydroxymatairesinol, 7-hydroxysecoisolariciresinol, arctigenin, conidendrin, coumestrol, dimethylmatairesinol, lariciresinol, lariciresinol-sesquilignan, medioresinol, pinoresinol/matairesinol, secoisolariciresinol-sesquilignan, secoisolariciresinol, todolactol A, sesamin, sesaminol, sesamol, sesamolinol, and syringaresinol [132,153]. The total lignan content in sunflower seeds is reported to be 0.4 mg/100 g [154]. Lignans, produced by the dimerization of two phenylpropanoids, represent a wide-ranging group of compounds. Some lignans, such as (+)- lyoniresinol in wine and spirits are known to impart bitterness [155], but the taste activity of lignans in sunflower seeds remains unknown.

In addition to phytosterols and lignans, sunflower seeds contain secondary metabolites such as saponin and cynarin, which exhibit anti-inflammatory and cholesterol-lowering effects [1]. Other bioactive compounds include sesquiterpene lactones such as costunolide, dehydrocostus lactone, 8-epixanthatin, and tomentosin [156] and the triterpene squalene, detected at a concentration of 0.51 µmol/kg [12]. Diterpenoids such as grandiflorolic acid, kaurenoic acid, and trachylobanoic acid have demonstrated significant anti-inflammatory activity in cell cultures and mouse models [16]. Crude sunflower oil contains 0.277 mg/100 g of carotenoids, mainly carotene, cryptoxanthin, lutein, and zeaxanthin as well as chlorophyll; however, these compounds are not known to be taste active [157,158]. Amakura et al. [158] identified novel compounds in sunflower seeds, including benzyl alcohol *β*-D-apiofuranosyl-(1→6)-β-D-(4-O-caffeoyl) glucopyranoside, methyl caffeate, methyl chlorogenate, and eriodictyol 5-O-β-D-glucoside. Furthermore, Jan et al. [159] reported the presence of caftaric acid; rosmanol; proanthocyanidin B1; and derivatives of sinapic acid, caffeic acid, gallic acid, caffeoylmalic acid, and quercetin.

Furthermore, sunflower seeds contain a small number of anti-nutritional compounds, including cyanide (4.026–4.175 mg/100 g), tannins (623–651 mg/100 g), and oxalates (98–113 mg/100 g). Although phytate reduces the bioavailability of minerals, it also exhibits antioxidative and cholesterol-lowering properties [2]. Tannins, which are known to impart bitterness and astringency to products such as red wine, are present in sunflower seeds; however, their contribution to the taste profile of sunflower products remains to be determined [160]. Many other phenols, such as various quinic acid derivatives [2], isoferulic acid, and 4-hydroxybenzoic acid [17], have been identified in sunflower seeds. However, not all are included in Table 6, as their taste activity remains largely unknown.

## 5. Discussion

Evidence suggests that flavor remains the primary determinant influencing food purchases [161]. Meeting these flavor expectations while addressing the global demand for sustainable protein sources poses a significant challenge, particularly considering the growing global population [162]. The exploration of plant-based protein sources and their side-stream products is therefore critical as potential solutions.

Sunflower-based food products offer a low-cost, plant-based alternative to animal-derived proteins, reducing the environmental footprint of protein production [13,163], which is consistent with global sustainability goals. Sunflowers, predominantly cultivated as oil crops and utilized as animal feed in the form of press cake after oil extraction, represent an underexplored opportunity as a protein alternative. The major limitation to their broader application in human nutrition is their undesirable flavor profile, a challenge commonly associated with plant proteins. The recent interest in utilizing sunflower meal/cake and protein concentrates as alternatives in plant-based food applications has further underscored the significance of addressing associated flavor challenges. These ingredients have been explored in a variety of products, such as cookies, snack bars, pasta, breads, and meat analogues [27,31,37,163,164,165,166].

To date, no comprehensive study has provided a systematic framework for mapping the complete flavor profile of sunflower seeds and their by-products. To address this gap, the present review consolidates the current knowledge on both volatile and non-volatile compounds in sunflower seeds that potentially influence their flavor. Volatile odor-active compounds are already well established as significant contributors to flavor perception [86]. Notably, although considerable research has focused on odor-active volatiles in sunflower oil, research on volatiles contributing to the aroma of sunflower seeds remains relatively limited. This focus on sunflower oil is logical, considering that sunflower is one of the most cultivated oil crops worldwide [167]. However, non-volatile taste-active compounds remain largely underexplored despite their critical role in determining sensory characteristics.

The flavor profile of sunflower seeds is shaped by a combination of macronutrients, including lipids, proteins, and carbohydrates as well as secondary metabolites such as polyphenols, minerals, and vitamins. Unique interactions among these components influence the overall flavor experience by contributing to different sensory characteristics. Lipids, primarily present as triacylglycerides, are the predominant components of sunflower seeds. Fresh lipids contribute minimal flavor, but their oxidation during storage or processing may lead to the release of free fatty acids, resulting in rancid and bitter off-notes. Such oxidation products are a well-documented source of undesirable tastes in plant-based protein isolates [168]. In contrast, proteins may impart desirable as well as undesirable flavors, as enzymatic hydrolysis leads to the release of umami-enhancing peptides and production of bitter-tasting hydrophobic peptides. However, their exact potential taste activity in sunflower-based products remains unknown. Carbohydrates, although less abundant in sunflower seeds, play a crucial role in flavor enhancement. They contribute sweetness and serve as the precursor for aroma-active compounds formed via the Maillard reaction during roasting, resulting in roasted and nutty flavor notes. To better understand complex interactions, it is also important to examine how macronutrients can modulate flavor perception at a molecular level. For example, proteins can bind aroma compounds through covalent as well as non-covalent (e.g., π,π, hydrophobic, and hydrogen) bonding interactions, while lipids primarily influence aroma retention via hydrophobic partitioning; both mechanisms can significantly affect flavor release and perception [169,170,171]. Additionally, carbohydrates may impact volatility by increasing matrix viscosity or through weak interactions such as hydrogen bonding and inclusion complex formation [171,172]. Within real food matrices, macronutrients coexist and may influence not only the behavior of flavor compounds but also one another’s functional and sensory contribution. These interactions may be governed by macronutrient structure and physicochemical properties, ultimately shaping the availability and perception of flavor compounds in the food matrix [171,172].

In addition to the contribution of macronutrients, the role of secondary metabolites that are abundant in sunflower also warrants attention. These compounds occur in both free and bound forms (e.g., esters, glycosides, or complexes), which significantly affects taste perception [1]. Moreover, it is well known that polyphenols interact at specific stages of various reaction cascades, further complicating their contribution to taste. Several phenolic compounds such as phenolic acids and flavonoids, which are abundant in sunflower seeds, are typically associated with a bitter and astringent taste. The intensity of the astringent sensation varies depending on the presence of other taste stimuli, highlighting the complexity of these interactions [173]. Moreover, minerals such as sodium and potassium contribute to saltiness, whereas calcium and magnesium impart bitterness or astringency. However, the extent of their contribution to sunflower flavor requires experimental validation. Similarly, the flavor impact of vitamins present in sunflower seeds remains unexplored, as the taste thresholds of vitamins have not yet been determined.

The intricate complexity of sunflower flavor arises from molecular interactions that influence both perceptual and cognitive processes. For instance, the intensity of taste perception from individual components may be modulated by the presence of others; for example, several bitter-tasting compounds at low concentrations may collectively activate various bitter receptors, leading to a bitter taste even if individual taste thresholds are not met [146]. This phenomenon is particularly noteworthy in plant-based proteins, as processing methods such as hydrolysis convert non-proteinogenic off-taste compounds into bitter derivatives, whereas proteinogenic compounds also contribute to the bitterness of protein hydrolysates [108]. Furthermore, cognitive processes, such as the integration and interpretation of multiple taste stimuli in the brain, add another layer of complexity. For example, “mixture suppression” reduces the perceived intensity of individual tastes in compound mixtures, as described by Keast and Breslin [70]. These dynamics may have synergistic effects, where compounds below their individual taste thresholds collectively enhance flavor perception, or suppressive effects, where certain compounds diminish the impact of other compounds.

A major limitation in existing research is the absence of integrated sensory and sensomics analyses. Although data on sunflower seed composition are available, the lack of systematic studies linking these compounds to actual sensory perception remains a bottleneck for advancing product development. By addressing these challenges, this review provides a foundational framework by consolidating existing data on known non-volatile compounds in sunflower seeds that may influence taste. However, experimental studies using advanced tools such as the sensomics approach are required to conclusively determine their direct contribution to sensory perception [43,44,174].

Furthermore, the effects of environmental factors, cultivar-specific differences, and stress conditions on the flavor profile of sunflower seeds need to be taken into consideration. Identifying and leveraging these variations is instrumental for optimizing sunflower products to meet both sensory and nutritional demands. Moreover, the choice of processing techniques, such as fermentation or enzymatic hydrolysis, should be guided by a deeper understanding of sunflower flavor dynamics. Identifying off-flavors is essential to selecting appropriate interventions for their mitigation while preserving nutritional quality. Therefore, selecting appropriate treatment methods on the basis of the intended application is critical for optimizing the sensory outcomes.

## 6. Conclusions

Sunflower has long been cultivated primarily as an oilseed crop, and scientific attention toward its flavor has largely focused on oil-based products. Of all sunflower-derived matrices, only cold-pressed and roasted sunflower oils have undergone systematic investigation using molecular sensory science (sensomics), allowing for the identification of key odorants through validated techniques. Recently, however, sunflower has also gained attention as a promising protein source in the development of plant-based foods and alternatives. This shift brings new opportunities but also significant flavor-related challenges, particularly in protein-rich matrices such as meal, press cake, concentrates, and isolates which remain largely uncharacterized from a sensory perspective. Although a few studies have noted the presence of off-putting aromas in these products, no systematic work has been conducted to identify their key odorants using the sensomics approach.

The knowledge gap is even more pronounced when it comes to taste. To date, no studies have been conducted to identify or validate taste-active compounds in sunflower protein products. As a consequence, the taste profile of sunflower protein remains poorly understood.

To fully utilize sunflower’s potential as a sustainable and nutritionally valuable protein source, it is critical to extend sensory research beyond oil and apply integrated sensomics methodologies to protein-based products. Identifying and confirming both odor- and taste-active compounds through reconstitution experiments, omission tests, and in vitro receptor assays while also considering matrix effects that modulate flavor perception will provide mechanistic understanding.

Once the key taste-active compounds are confirmed through these methods and their sensory contributions are well understood, it becomes possible to implement precise interventions to improve flavor. As demonstrated by Mittermeier-Kleßinger et al. [108], strategies such as controlled fermentation, selection of clean protein isolates, and genotype optimization can be employed to minimize undesirable taste impressions and enhance overall acceptability. These approaches, framed within the concept of delivering a full flavor experience, enable the development of targeted, scalable solutions that can address the sensory complexity and technological constraints of sunflower-based ingredients while unlocking their full potential in modern plant-based foods to match consumer acceptance.

## Figures and Tables

**Table 1:** Nutrient composition of dried sunflower kernels according to the United States Department of Agriculture [4].

Parameter	Content (g/100 g Kernels)
Moisture	4.73
Protein	20.8
Fat	51.5
Carbohydrates	20.0
Fiber	8.6
Ash	3.02

**Table 2:** The main volatile compounds in sunflower products with odor thresholds and sensory attributes.

	Compound(s)	Reported Concentrations (µg/kg)	Odor Threshold (µg/kg)	Odor Description	Method ^a,b,d,e^
**Raw and roasted seeds**	*α*-pinene	7570 ^f^, 21,850 ^r^	6 ^a,e^	pine	HS-SPME-GC-MS, multivariate statistical analysis
	*β*-pinene	760 ^f^, 3000 ^r^	140 ^a^	woody, pine-like	
	octane	2670 ^f^	**/**	/	
	furfural	950 ^f^, 8180 ^r^	3000 ^e^	almond, sweet	
	hexanal	1350 ^f^, 8490 ^r^	479 ^e^	green, fatty	
	γ-butyrolactone	1150 ^f^, 3190 ^r^	1000 ^e^	creamy	
	2-methylbutanal	540 ^f^, 2110 ^r^	23 ^e^	malty, almond	
	2,5-dimeththylpyrazine	200 ^f^, 10,190 ^r^	800 ^e^	roasty, cocoa	
	2,3-dimethylpyrazine	550–1300 ^r^	100 ^e^	nut, peanut, cocoa,	
	2-ethyl-3- methylpyrazine	140 ^f^ 3890 ^r^	20 ^e^	nutty, cereal like	
	2-ethyl-3,5-dimethylpyrazine	2090 ^r^	7.5 ^e^	nutty	
**Cold-pressed oil**	*α*-pinene	11,145 ^a^–94,890 ^b^	6 ^a^	woody, pine-like	Dynamic HS-GC-MS; SPME-GC-MS, QDA; Molecular sensory science/Sensomics
	*β*-pinene	4068 ^a^	140 ^a^	woody, pine-like	Dynamic HS-GC-MS; Molecular sensory science
	sabinene	/	980 ^c^	woody, citrus-like	Dynamic HS-GC-MS
	limonene	/	2100 ^c^	lemon, citrus	
	hexanal	541 ^a^	73 ^a^	green, grassy	Dynamic HS-GC-MS; Molecular sensory science/Sensomics
	3-methyl-1-butanol	200–480 ^b^	100 ^b^	nutty, fruity	SPME-GC-MS, QDA
	linalool	56 ^a^	6 ^a^	citrus, fruity	Molecular sensory science/Sensomics (HS-SPME GC-O-MS, SAFE, AEDA, OAV, GC-O, recombination model)
	octanal	125 ^a^	56 ^a^	fruity, green	
	*α*-phellandrene	36 ^a^	40 ^a^	citrus, sweet, peel	
	(*E*)-2-octenal	69 ^a^	61 ^a^	fatty, floral	
**Roasted oil**	2-methylbutanal	6726 ^a^	34 ^a^	roasted, malty	Molecular sensory science/Sensomics (HS-SPME GC-O-MS, SAFE, AEDA, OAV, GC-O, recombination model)
	3-methylbutanal	714 ^a^	15 ^a^	fatty, almond	
	2,6-dimethylpyrazine	2329 ^a^	20 ^a^	nutty, roasted, coffee	
	2,5-dimethylpyrazine	12,228 ^a^	200 ^a^	nutty, potato	
	2,3-dimethylpyrazine	238 ^a^	8 ^a^	nutty, popcorn	
	2,3-dimethyl-5-ethylpyrazine	213 ^a^	100 ^a^	roasted, nutty, sweet	
	2,3-pentanedione	1456 ^a^	50 ^a^	buttery, sweet, spicy	
	2-pentylfuran	1332 ^a^	130 ^a^	buttery, caramel	
	1-pentanol	693 ^a^	470 ^a^	bread-like, sweet	

^a^—Yin et al. [79], ^b^—Bendini et al. [86], ^c^—Li et al. [89], ^d^—Bocci et al. [85], ^e^—Guo et al. [81]. ᶠ—Concentration in raw sunflower seeds, ʳ—highest concentration in roasted sunflower seeds. Relative concentration values for ᶠ and ʳ are taken from Guo et al. [81]. Odor descriptions are taken from corresponding literature where the data were reported. Threshold values vary between studies, which may be due to differences in matrix, methodology, or the population used for sensory determination.

**Table 3:** Fatty acids in sunflower seeds and their potential contribution to taste.

Name	Content (µmol/kg) *^R^*_1_	Taste	Taste Threshold (µmol/kg) *^R^*_2_	DoT
*α*-Linolenic acid	~539–1077	Scratchy, bitter	~189 ^a^, ~277 ^b^	~1.9–3.9
Linoleic acid	~17,508–26,422	Scratchy, bitter	~270 ^a^, ~1810 ^b^	~9.7–14.6
Oleic acid	~9983–29,631	Scratchy, bitter	~203 ^a^, ~2180 ^b^	~4.6–13.6
Palmitic acid	~2067–3003	Scratchy, bitter	~1002 ^a^, ~1546 ^b^	~1.3–1.9
Stearic acid	~949–1582	Scratchy, bitter	~645 ^a^, ~726 ^b^	~1.3–2.2

^a^—threshold for scratchy perception, ^b^—threshold for bitter taste. *^R^*_1_—data from Thepthanee et al. [103]; *^R^*_2_—data from Günther-Jordanland et al. [102]. DoT—dose-over-threshold factor.

**Table 4:** Amino acids in sunflower seeds with potential taste activity.

Name	Content (µmol/kg) *^R^_1_*	Taste	Taste Threshold (µmol/kg) *^R^_2_*	DoT
Alanine	~2110.4	Sweet	~12,000	<1
Arginine	/	Bitter	~75,000	
Aspartic acid	~255.53	Umami	~600	<1
Glutamic acid	~1229.56	Umami	~1100	1.1
Glycine	~1810.93	Sweet	~25,000	<1
Histidine	/	Bitter	~45,000	
Isoleucine	/	Bitter	~10,000	
Leucine	~383.84	Bitter	~11,000	<1
Lysine	/	Bitter	~80,000	
Methionine	/	Sweet	~5000	
Phenylalanine	/	Bitter	~45,000	
Proline	/	Sweet	~25,000	
Serine	/	Sweet	~25,000	
Threonine	/	Sweet	~35,000	
Tyrosine	/	Bitter	~4000	
Valine	/	Bitter	~30,000	

*^R^*_1_—data from Petraru et al. [117]; *^R^*_2_—data from Hillmann et al. [120]. DoT—dose-over-threshold factor. In the referenced study, amino acids were extracted, derivatized, and analyzed using GC-MS. Analytical conditions and derivatization protocols may vary across studies, potentially affecting the reported amino acid content.

**Table 5:** Minerals with potential taste effects in sunflower seeds.

Name	Content (µmol/kg) *^R^*_1_	Taste	Taste Threshold (µmol/kg) *^R^*_2_	DoT
Calcium	~19,461–28,942	Bitter, astringent	~6200	~3.1–4.7
Magnesium	~124,254	Bitter, astringent	~6400	~19.4
Potassium	~168,030	Salty	~13,000	~12.9
Sodium	<~1087	Salty	~3900	<1

*^R^*_1_—data from USDA report [126]; *^R^*_2_—data from Dirndorfer et al. [46]. DoT—dose-over-threshold factor.

**Table 6:** Phenolic acids in sunflower seeds and their taste qualities and potential flavor contribution.

Name	Content (µmol/kg) *^R^*_1_	Taste	Taste Threshold (µmol/kg) *^R^*_2_	DoT
Caffeic acid	~142–1482 ^a^	Astringent	~72	~2–20
*p*-Coumaric acid	~15.2 ^b^	Astringent	~139	<1
Ferulic acid	~87–639	Astringent	~67	~1.2–9.5
Gallic acid	~65.8 ^b^	Astringent	~292	<1
*p*-Hydroxybenzoic acid	/	Astringent	~665	
Protocatechuic acid	~329.6 ^b^	Astringent	~206	~1.6
Rosmarinic acid	~233–391	Bitter	~102.6	~2–3.8
Sinapic acid	~69.6 ^b^	Astringent	~693	<1
Syringic acid	/	Astringent	~263	
Vanillic acid	/	Astringent	~315	

^a^—dry matter, ^b^—free acids. *^R^*_1_—data from Khurana et al. [2], Paja̧k et al. [7], Žilic et al. [137] and Pedrosa et al. [135]. *^R^*_2_—data from Günther-Jordanland et al. [102], Hofmann et al. [141] and Gracia et al. [140]. DoT—dose-over-threshold factor. Quantification methods, analytical conditions, and sample matrices in the referenced studies vary. Such variation may affect the reported concentrations, thresholds, and DoT values.

**Table 7:** Flavonoids in sunflower seeds and their potential roles in taste perception.

Name	Content (µmol/kg) *^R^*_1_	Taste	Taste Threshold (µmol/kg) *^R^*_2_	DoT
Apigenin	~11	Bitter	/	
Catechin	/	Bitter, astringent	~800, 410	
Coumestrol	~0.004 ^a^	Bitter	~250”	<1
Daidzein	~0.094 ^a^	Bitter	~500”	<1
Epicatechin	/	Bitter, astingent	~1000, 930	
Epicatechin gallate	/	Astringent	~260	
Epigallocatechin	/	Astringent	~520	
Epigallocatechin gallate	/	Astringent	~190	
Formononetin	~0.026 ^a^	Bitter	~500”	<1
Genistein	~0.074 ^a^	Bitter	~4–8”	<1
Glycetein	~0.018 ^a^	Bitter	~500”	<1
Kaempferol	~1.75	Bitter, astringent	~69.87	<1
Luteolin	/	Bitter	/	
Myricetin	~131.9–194.8	Bitter, astringent	~31.42	~4.2–6.2
Quercetin	~19 ^b^	Bitter, astringent	~33.09	<1
Rutin	/	Astringent	~0.00115	

^a^—in wet weight; “—measured in vitro, ^b^—free and bonded ***^R^***_1_—data from Pająk, P. et al. [7], Žilić, S. et al. [137], Thompson et al. [149]. ***^R^_2_***—data from Scharbert et al. [71], Stark, et al. [128], Roland et al. [150], Gonzalo-Diago et al. [151]. DoT—dose-over-threshold factor. Quantification methods, analytical conditions, and sample matrices in the referenced studies vary. Such variation may affect the reported concentrations, thresholds, and DoT values.

## Data Availability

No new data were created or analyzed in this study. Data sharing is not applicable to this article.
